# Association of HIF1-α gene polymorphisms with advanced non-small cell lung cancer prognosis in patients receiving radiation therapy

**DOI:** 10.18632/aging.202542

**Published:** 2021-02-17

**Authors:** Yan Zhang, Jian Wang, Zhanzhan Li

**Affiliations:** 1 Department of Pathology, The Second Affiliated Hospital of Zhengzhou University, Zhengzhou 450014, Henan Province, China; 2 Department of Oncology, The Second Affiliated Hospital of Zhengzhou University, Zhengzhou 450014, Henan Province, China; 3 Department of Oncology, Xiangya Hospital, Central South University, Changsha 410008, Hunan Province, China

**Keywords:** non-small cell lung cancer, gene polymorphisms, prognosis, survival

## Abstract

We investigated the association between single nucleotide polymorphisms (SNPs) in the *HIF1A* gene and the prognosis of advanced non-small cell lung cancer (NSCLC) patients undergoing radiation therapy. Patient overall survival (OS) and progression-free survival (PFS) were analyzed. The rs11549465 TT genotype was associated with poor PFS (P<0.001) and OS (*P*=0.001). The rs2057482 TT genotype was also associated with poor PFS (*P*=0.002) and OS (*P*=0.007). Stratified analyses revealed that these associations occurred in patients with a smoking history, squamous cell carcinoma, and stage IIIA disease, as well as those receiving radiation therapy a radiation dose of ≥70 Gy. We found associations between SNPs and PFS but not OS in patients without a smoking history, other histological types, and stage IIIB disease, as well as those undergoing chemoradiotherapy with a radiation dose of <70 Gy. No associations were observed between rs11549467 or rs110873142 and NSCLC prognosis. These results suggest that *HIF1A* polymorphisms can be used as independent prognostic biomarkers for NSCLC patients receiving radiation therapy.

## INTRODUCTION

Lung cancer is the leading cause of cancer-related deaths, and its incidence is rapidly increasing [1, 2]. Non-small cell lung cancer (NSCLC) accounts for more than 75% of all lung cancers [3]. Radiation therapy is used to treat malignant tumors, including locally advanced lung cancer [4]. However, the long-term prognosis of lung cancer remains poor, with a 5-year survival rate of 5%-25% as most lung tumors are diagnosed at an advanced stage [5]. Many prognostic biomarkers have been explored in NSCLC, including tumor volume, stage, differentiation, and invasion [6]. With the development of novel treatment technologies and personalized treatment technologies, it is imperative to identify robust prognostic biomarkers at the cellular and molecular levels. Numerous gene polymorphisms are associated with cancer risk and prognosis [7–10]. Therefore, genetic markers may help guide clinical decision-making and improve NSCLC prognosis.

Hypoxia-inducible factor 1 (HIF-1) is a heterodimer composed of a 120-kD subunit (HIF-1α) and a 91-94-kD subunit (HIF-1β). HIF-1 expression and activity are tightly regulated by cellular oxygen levels, and HIF-1 is the master regulator of hypoxia-induced gene expression. HIF-1 plays a key role in tumor angiogenesis, cancer cell growth, proliferation, apoptosis, energy metabolism, and tumor metastasis. Recently, several *HIF1* single nucleotide polymorphisms (SNPs) have been shown to increase lung cancer risk. For example, Pura et al. reported that the frequency of the *HIF1A C1772T* variant was higher in lung cancer patients with p53 mutations and 1p/19q loss of heterozygosity [11]. Additionally, HIF-1α *1772 C/T (P582S)* and *1790 G/A (A588T)* polymorphisms are associated with NSCLC risk [12]. A recent meta-analysis showed that HIF-1α rs11549465 and rs11549467 polymorphisms increased the lung cancer risk in Asian populations [13]. Mounting evidence suggests that the C2028T polymorphism in *HIF1* exon 12 and the dinucleotide repeat polymorphism in intron 13 influence HIF-1 expression in lung cancer; thus, functional *HIF1* polymorphisms may significantly affect lung cancer development by causing genomic instability. In a small cohort study, the *1772T>C* polymorphism has been demonstrated to influence lung cancer prognosis and chemotherapy response [14]. The study also indicated that the expression levels of HIF-1α were associated with lymph node metastasis in breast cancer [15] and with disease-free survival and overall survival (OS) in rectal cancer [16]. In this study, we aimed to identify the *HIF1A* SNPs associated with the survival of patients with advanced NSCLC undergoing radiation therapy.

## RESULTS

### Patient characteristics

The study cohort consisted of 512 NSCLC patients receiving radiation therapy. Complete clinical characteristics and follow-up data were obtained for all patients. The median follow-up time was 25.02 months (range, 4.05-102.39 months). A total of 284 (55.5%) patients died, and the disease progressed in 192 patients for a median of 17.3 months. The mean patient age was 57.8 ± 8.6 years. The cohort consisted of 279 (54.5%) men and 233 (45.5%) women, with 188 patients being overweight (BMI ≥24). Smokers and drinkers represented 57.2% (n=293) and 48.8% (n=250) of the cohort, respectively, and 263 patients had a Karnofsky performance status (KPS) of <80. Squamous cell carcinoma (SCC) was diagnosed in 192 patients, and adenocarcinoma (ADC) or other types were diagnosed in 320 patients. Among all patients, 44.3% (n=227) and 55.7% (n=285) had stage IIIA and stage IIIB tumors, respectively. Chemotherapy was combined with radiotherapy for 207 (40.4%) patients. For radiotherapy, 263 (51.4%) patients underwent IMRT, and 249 (48.6%) received 3D-CRT.

### The association between clinical characteristics and prognosis

The association between clinical characteristics and NSCLC patient survival (OS and PFS) are shown in Table 1. We found that age was not associated with PFS (*P*=0.643) or OS (*P*=0.660). Although gender was not associated with PFS, men had slightly worse OS compared to women (HR=1.27, 95% CI: 1.00-1.60, *P*=0.048). Smoking was associated with PFS and OS. Notably, smokers had worse PFS (HR=2.48, 95% CI: 1.79-3.42, *P*<0.001) and OS (HR=1.55, 95% CI: 1.21-1.98, *P*<0.001) than non-smokers. The TNM (Tumor Node Metastasis) stage was also associated with NSCLC prognosis. Patients with stage IIIB disease had a shorter duration of PFS (HR=2.17, 95% CI:1.59-2.95, *P*<0.001) and OS (HR=1.72, 95% CI:1.35-2.21, *P*<0.001) than patients with stage IIIA disease. Furthermore, patients receiving a radiation dose of <70 Gy had a poorer PFS (HR=1.74, 95% CI:1.31-2.32, *P*<0.001) and OS (HR=1.31, 95% CI:1.04-1.66, *P*=0.022) than patients receiving a dose of ≥70 Gy. Drinking (*P*=0.595 for PFS, *P*=0.992 for OS), KPS (*P*=0.873 for PFS, *P=*0.249 for OS), histological type (*P=*0.5780 for PFS, *P*=0.227 for OS), and chemotherapy (*P*=0.132 for PFS, *P*=0.074 for OS) did not affect patient survival.

**Table 1:** Association between clinical characteristics and progression-free survival and overall survival in patients with NSCLC.

**Parameters**	**Category**	**Progression-free survival**				**Overall survival**			
		**MST**	**Event/Total**	**HR (95%CI)**	***P***^a^	**MST**	**Event/Total**	**HR (95%CI)**	***P***^a^
Age	<60	58.5	70/193	1.00		46.3	105/193	1.00	
	≥60	53.3	122/319	1.07(0.80-1.44)	0.643	48.2	179/319	1.06(0.83-1.34)	0.660
Sex	Female	56.2	90/233	1.00		49.9	122/233	1.00	
	Male	54.2	102/279	1.01(0.76-1.34)	0.957	44.7	162/319	1.27(1.00-1.60)	0.048
Body mass index	<24	54.4	127/324	1.00		45.2	181/324	1.00	
	≥24	54.6	65/188	0.85(0.63-1.15)	0.289	49.7	103/188	0.93(0.73-1.19)	0.930
Smoking	No	70.0	49/219	1.00		56.3	95/219	1.00	
	Yes	43.7	143/293	2.48(1.79-3.42)	<0.001	40.9	189/293	1.55(1.21-1.98)	0.001
Drinking	No	55.6	95/262	1.00		47.7	143/262	1.00	
	Yes	54.9	97/250	1.08(0.81-1.43)	0.595	46.3	141/250	0.99(0.79-1.25)	0.992
KPS	≥80	55.8	95/249	1.00		47.9	130/249	1.00	
	<80	54.6	97/263	1.02(0.77-1.36)	0.873	46.2	154/263	1.15(0.91-1.45)	0.249
Histology	SCC	54.8	73/192	1.00		44.1	114/192	1.00	
	ADC and other	55.0	119/320	0.96(0.72-1.28)	0.780	47.8	170/320	0.86(0.68-1.10)	0.227
TNM stage	IIIA	64.1	58/227	1.00		58.7	93/227	1.00	
	IIIB	48.3	134/285	2.17(1.59-2.95)	<0.001	39.9	191/285	1.72(1.35-2.21)	<0.001
Chemotherapy	No	57.8	108/305	1.00		48.0	161/305	1.00	
	Yes	48.6	84/207	1.25(0.94-1.66)	0.132	44.9	123/207	1.24(0.98-1.57)	0.074
Radiation technique	IMRT	53.0	99/263	1.00		47.9	145/263	1.00	
	CRT and other	55.1	93/249	0.96(0.73-1.28)	0.799	46.2	139/249	0.95(0.75-1.19)	0.634
Dose	≥70Gy	63.2	80/269	1.00		49.6	134/269	1.00	
	<70Gy	44.3	112/243	1.74(1.31-2.32)	<0.001	44.1	150/243	1.31(1.04-1.66)	0.022

*P*^a^*:* P value for univariate cox regression; MST: mean survival time; KPS: Karnofsky Performance Status; SCC, squamous cell carcinoma; ADC, adenocarcinoma; IMRT, Intensity Modulated Radiation Therapy; CRT, three-dimensional conformal radiotherapy.

### The association between HIF1A gene polymorphisms and survival of NSCLC patients receiving radiation therapy

**Figure 1:**
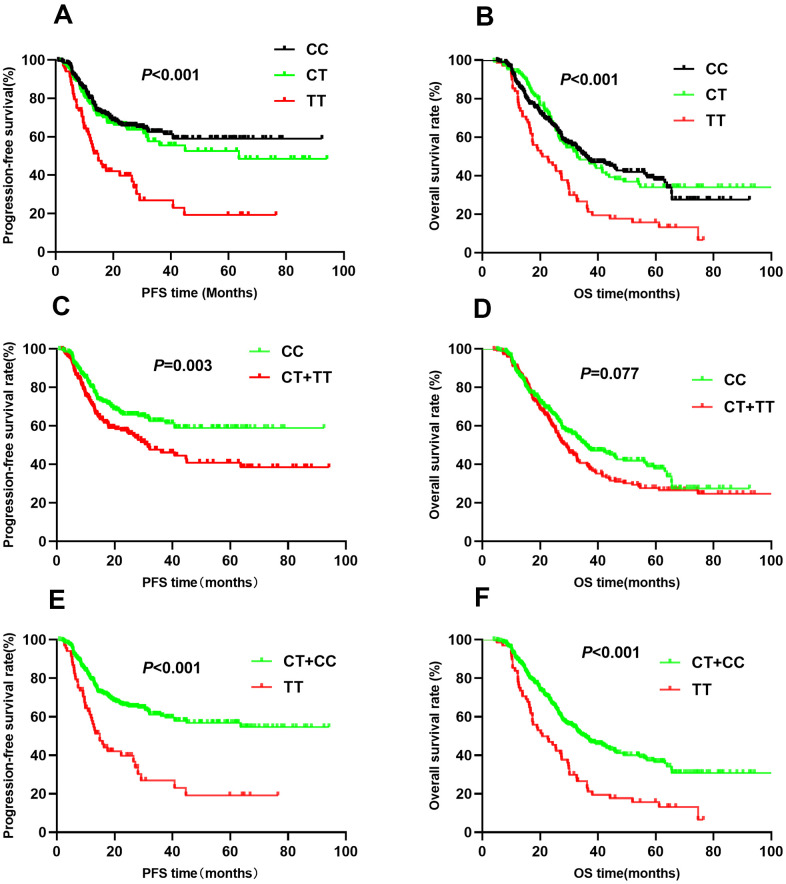
Kaplan-Meier survival curve analysis showing progression-free survival ((**A**) CC vs CT vs TT, (**C**) CT+TT vs CC, (**E**) TT vs CT+CC) and overall survival ((**B**) CC vs CT vs TT, (**D**) CT+TT vs CC, (**F**) TT vs CT+CC) of NSCLC patients with HIF1-alpha rs11549465.

**Figure 2:**
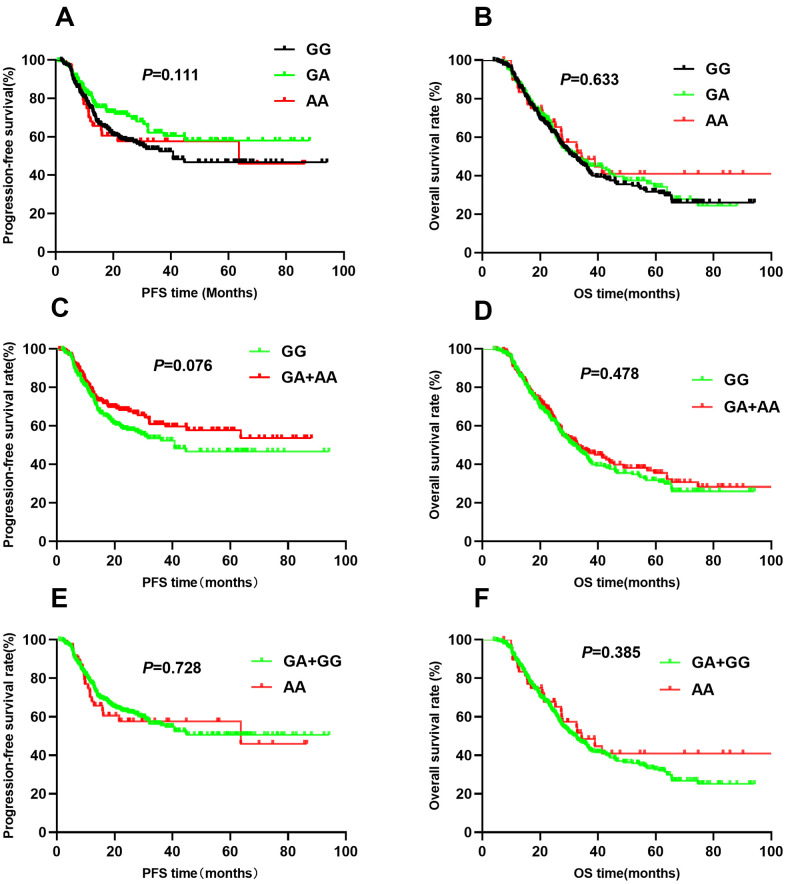
Kaplan-Meier survival curve analysis showing progression-free survival ((**A**) GG vs GA vs AA, (**C**) GA+AA vs GG, (**E**) AA vs GA+GG) and overall survival (**B**) GG vs GA vs AA, (**D**) GA+AA vs GG, (**F**) AA vs GA+GG) of NSCLC patients with HIF1-alpha rs11549467.

The association between *HIF1A* gene polymorphisms and NSCLC patient survival (PFS and OS) were assessed using Kaplan-Meier analysis and multivariate Cox regression analysis (Table 2). Of the four SNPs (rs11549465, rs11549467, rs2057482, and rs10873142), two (rs11549467 and rs10873142) were not associated with patient survival. Interestingly, rs11549465 was associated with NSCLC prognosis. Patients with CT (median survival time [MST]: 56.2 months) and TT (MST: 27.4 months) genotypes had a shorter duration of PFS (*P*<0.001, Figure 1A) and OS (MST: 47.7 for CC, 52.1 for CT, 29.3 for TT, *P*<0.001) compared to patients with CC genotypes (MST: 60.8, Figure 1B). After adjusting for potential confounding factors, multivariate cox regression indicated that TT genotype was associated with poorer PFS (HR=2.07, 95% CI: 1.42-3.01, *P*<0.001) and OS (HR=1.63, 95% CI: 1.18-2.24, *P*=0.003). Patients with CT or TT polymorphisms had shorter median PFS compared to those with CC polymorphisms (47.7 vs. 60.8, *P*=0.003, Figure 1C). The median OS was similar between patients with CT+TT and CC polymorphisms (44.8 vs. 47.7, *P*=0.077, Figure 1D). Patients with CT+TT alleles had a lower PFS (HR=1.39, 95% CI: 1.03-1.86, *P*=0.029) than those with a CC genotype, although the OS was similar between the 2 groups (HR=1.16, 95% CI: 0.91-1.47, *P*=0.239). Patients with the TT genotype had a poorer PFS (MST: 27.4 vs. 59.7, *P*<0.001, Figure 1E) and OS (MST: 29.3 vs. 51.4, *P*<0.001, Figure 1F) compared to those with the CC+CT genotype. The TT genotype was associated with an increased risk of adverse outcomes (PFS: HR=2.01, 95% CI: 1.14-2.85, *P*<0.001; OS: HR=1.66, 95% CI: 1.22-2.24, *P*=0.001). For rs11549467, there were no significant differences in PFS (GG vs. GA vs. AA: 52.7 vs. 58.4 vs. 51.7, *P*=0.111, Figure 2A) or OS (GG vs. GA vs. AA: 45.0 vs. 44.6 vs. 55.4, *P*=0.633, Figure 2B) compared with the other three genotypes. Moreover, we found no differences in PFS (*P*=0.076, Figure 2C) or OS (*P*=0.478, Figure 2D) between the GA+AA and GG groups. Multivariate Cox regression analyses confirmed similar results for AA vs. GG+GA (PFS: *P*=0.728, Figure 2E; OS: *P*=0.385, Figure 2F).

**Table 2:** Associations of HIF1-alpha gene with PFS and OS in patients with NSCLC.

**SNP**	**Progression-free survival**					**Overall survival**				
	**Event/No.**	**MST**	**P**^a*^	**Adjusted HR (95%CI)**	**P**^b*^	**Event/No.**	**MST**	**P**^a*^	**Adjusted HR (95%CI)**	**P**^b*^
rs11549465										
CC	96/298	60.8		1.00		151/298	47.7		1.00	
CT	52/146	56.2		1.09(0.77-1.54)	0.621	76/146	52.1		0.95(0.72-1.26)	0.726
TT	44/68	27.4	<0.001	2.07(1.42-3.01)	<0.001	57/68	29.3	<0.001	1.63(1.18-2.24)	0.003
Trend^#^										
CC	96/298	60.8		1.00		151/298	47.7		1.00	
CT+TT	96/214	47.7	0.003	1.39(1.03-1.86)	0.029	133/214	44.8	0.077	1.16(0.91-1.47)	0.239
CC+CT	148/444	59.7		1.00		227/444	51.4		1.00	
TT	44/68	27.4	<0.001	2.01(1.41-2.85)	<0.001	57/68	29.3	<0.001	1.66(1.22-2.24)	0.001
rs11549467										
GG	124/311	52.7		1.00		171/311	45.0		1.00	
GA	48/152	58.4		0.76(0.54-1.06)	0.110	89/152	44.6		1.02(0.79-1.33)	0.856
AA	20/49	51.7	0.111	1.15(0.71-1.87)	0.565	24/49	55.4	0.633	0.92(0.59-)1.42	0.697
Trend^#^										
GG	124/311	52.7		1.00		171/311	45.0		1.00	
GA+AA	68/201	56.6	0.076	0.84(0.62-1.14)	0.264	113/201	49.8	0.478	1.00(0.78-1.28)	0.999
GG+GA	172/463	55.8		1.00		260/463	45.3		1.00	
AA	20/49	51.7	0.728	1.26(0.78-2.02)	0.340	24/49	55.4	0.385	0.91(0.59-1.39)	0.909
rs2057482										
CC	100/314	61.3		1.00		161/314	48.4		1.00	
CT	68/161	48.9		1.32(0.97-1.81)	0.078	90/161	49.3		1.03(0.79-1.33)	0.855
TT	24/37	19.8	<0.001	2.05(1.30-3.23)	0.002	33/37	28.5	0.002	1.71(1.16-2.51)	0.007
Trend^#^										
CC	100/314	61.3		1.000		161/314	48.4		1.00	
CT+TT	92/198	44.7	0.005	1.46(1.09-1.94)	0.010	123/198	44.2	0.254	1.14(0.90-1.45)	0.270
CC+CT	168/475	58.0		1.00		251/475	50.4		1.00	
TT	24/37	19.8	<0.001	1.84(1.19-2.86)	0.007	33/37	28.5	<0.001	1.69(1.16-2.46)	0.006
rs10873142										
TT	119/288	52.0		1.00		177/288	44.6		1.00	
TC	61/172	57.5		0.80(0.59-1.10)	0.167	83/172	49.5		0.82(0.63-1.06)	0.130
CC	12/52	57.1	0.076	0.53(0.29-)0.96	0.037	24/52	51.8	0.081	0.68(0.44-1.04)	0.078
Trend^#^										
TT	119/288	52.0		1.00		177/288	44.6		1.00	
TC+CC	73/224	59.7	0.080	0.74(0.55-1.02)	0.054	107/224	50.9	0.038	0.78(0.61-1.00)	0.045
TT+TC	180/460	54.5		1.00		260/460	47.1		1.00	
CC	12/52	57.1	0.044	0.57(0.31-1.03)	0.063	24/52	51.8	0.102	0.73(0.45-1.11)	0.139

*P^a^, Log-rank P; *P*^b^, multivariate Cox regression; MST, mean survival time.

^#^Trend: the prognosis showed an increased or decreased changed with the number of risk allele.

**Figure 3:**
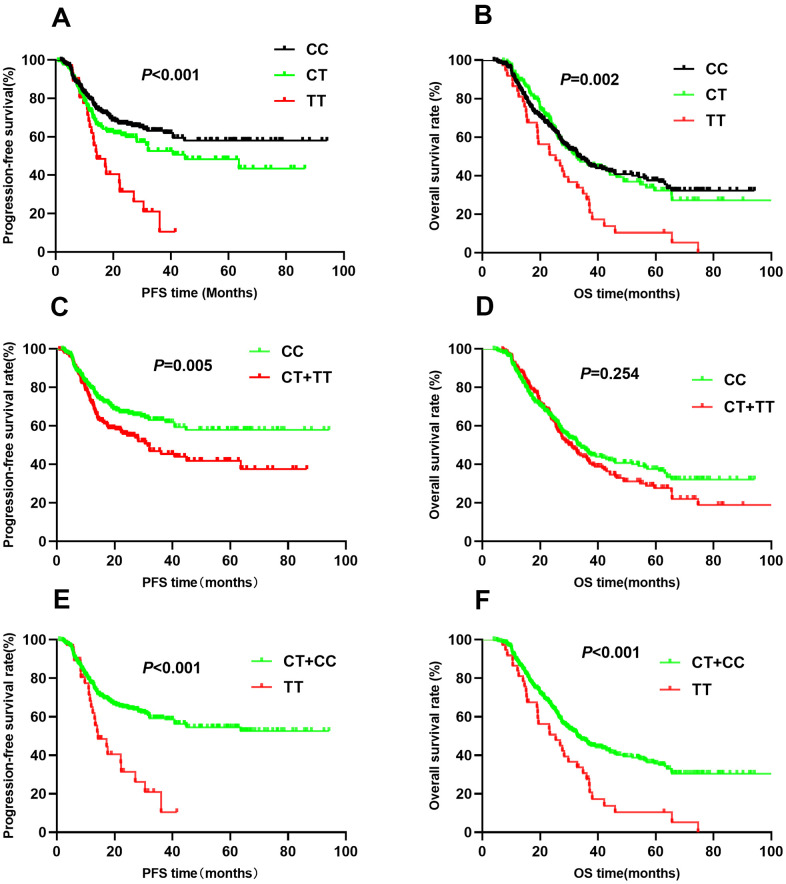
Kaplan-Meier survival curve analysis showing progression-free survival ((**A**) CC vs CT vs TT, (**C**) CT+TT vs CC, (**E**) TT vs CT+CC) and overall survival ((**B**) CC vs CT vs TT, (**D**) CT+TT vs CC, (**F**) TT vs CT+CC) of NSCLC patients with HIF1-alpha rs2057482.

The rs2057482 SNP was associated with poor NSCLC patient survival. Compared to patients with CC genotypes, those with CT and TT genotypes had a shorter median PFS (CC vs. CT vs. TT: 61.3 vs. 48.9 vs. 19.8, *P*<0.001, Figure 3A) and OS (CC vs. CT vs. TT: 48.4 vs. 49.3 vs. 28.5, *P*<0.001, Figure 3B). Multivariate Cox regression analyses indicated that the TT genotype was associated with a poor PFS (HR=2.05, 95% CI: 1.30-3.23, *P*=0.002) and OS (HR=1.71, 95% CI: 1.16-2.51, *P*=0.007). Trend analysis indicated that the domain effect model (CT+TT) was associated with a shorter median PFS (44.7 vs. 61.3, *P*=0.005, Figure 3C), whereas the median OS was similar between the CT+TT and CC groups (44.2 vs. 48.4, *P*=0.254, Figure 3D). Patients with CT+TT genotypes had worse PFS (HR=1.46, 95% CI: 1.09-1.94, *P*=0.010) than those with the CC genotype; However, the OS was similar (HR=1.14, 95% CI: 0.90-1.45, *P*=0.70). Kaplan-Meier analysis indicated that patients with the TT genotype had worse PFS (MST: 19.8 vs. 58.0, *P*<0.001, Figure 3E) and OS (MST: 28.5 vs. 50.4, *P*<0.001, Figure 3F) than those with the CC+CT genotypes. Cox regression analyses confirmed that the TT genotype increased the risk of adverse outcomes (PFS: HR=1.84, 95% CI: 1.19-2.86, *P*=0.007; OS: HR=1.69, 95% CI: 1.16-2.46, *P*=0.006).

**Figure 4:**
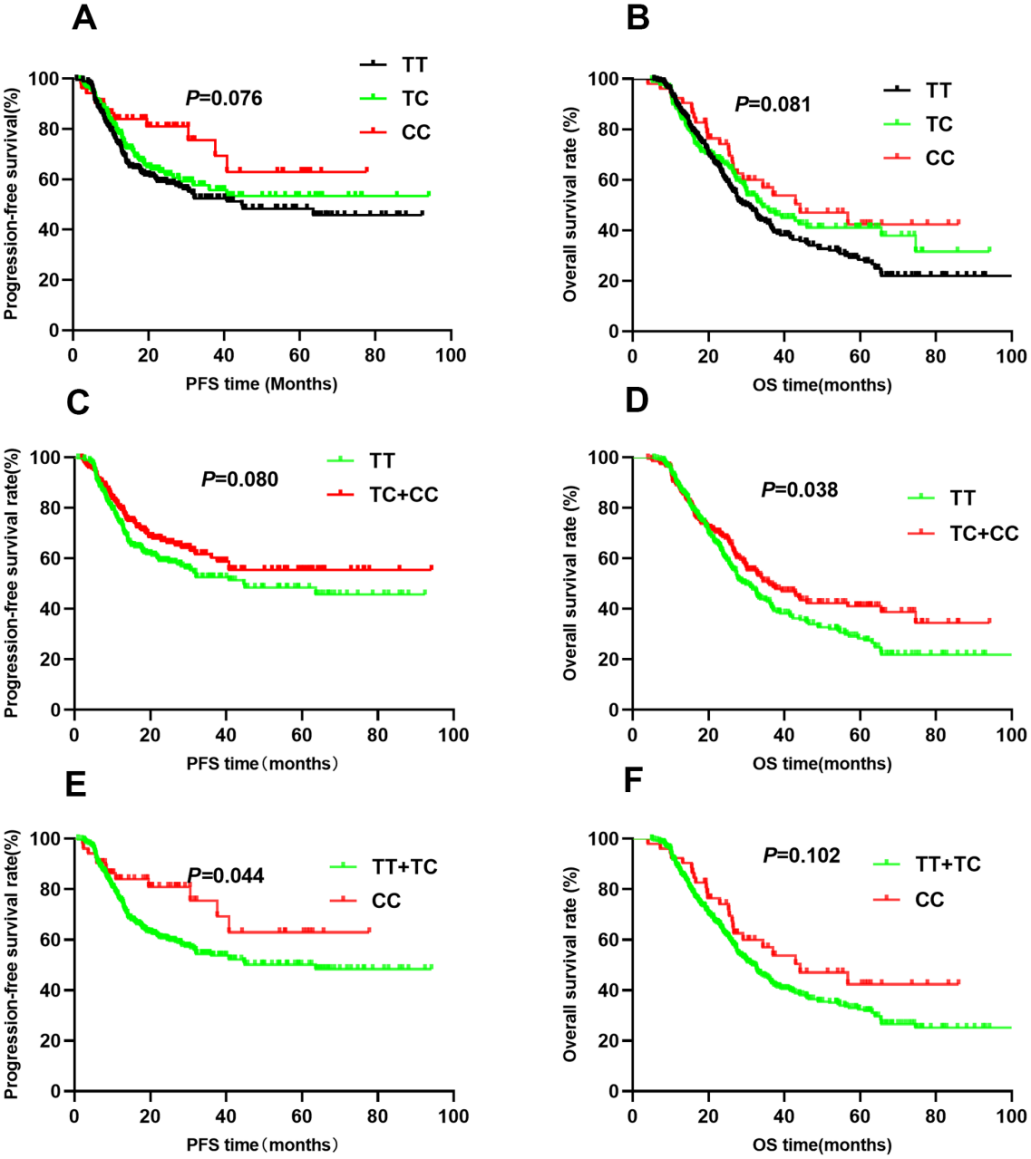
Kaplan-Meier survival curve analysis showing progression-free survival ((**A**) CC vs CT vs TT, (**C**) CT+TT vs CC, (**E**) TT vs CT+CC) and overall survival ((**B**) CC vs CT vs TT, (**D**) CT+TT vs CC, (**F**) TT vs CT+CC) of NSCLC patients with HIF1-alpha rs10873142.

For rs10873142, no differences were observed in PFS (TT vs. TC vs. CC: 52.0 vs. 57.5 vs. 57.1, *P*=0.076, Figure 4A) or OS (TT vs. TC vs. CC: 44.6 vs. 59.5 vs. 51.8, *P*=0.081, Figure 4B) among the different genotypes. Patients with TC+CC genotypes had a similar PFS as those with the TT genotype (*P*=0.080, Figure 4C). Trend analysis indicated a weak association between the TC+CC genotypes and OS (*P*=0.038, Figure 4D). Additionally, the CC genotype was associated with PFS (*P*=0.044, Figure 4E) but not OS (*P*=0.102, Figure 4F). Multivariate Cox regression analyses showed that genotypes were associated with PFS. However, the TC+CC genotype was weakly associated with OS (HR=0.78, 95% CI: 0.61-1.00, *P*=0.045). To correct for multiple comparisons, we calculated the false-positive probability with a prior probability of 0.01 to detect an HR of 1.2 or 0.83. These results are presented in Table 3. The associations of all four SNPs remained significant with a prior FPRP of 0.1.

**Table 3:** False-positive reports probability values for associations between gene and survival outcomes.

**SNP**	**Progression-free survival**					**Overall survival**				
	**HR (95%CI)**	***P***^a^	**Prior probability**			**HR (95%CI)**	***P***^a^	**Prior probability**		
			0.2	0.1	0.01			0.2	0.1	0.01
rs11549465										
CT vs. CC	1.09(0.77-1.54)	0.621	0.090	0.182	0.710	0.95(0.72-1.26)	0.726	0.041	0.087	0.513
TT vs. CC	2.07(1.42-3.01)	<0.001	0.118	0.231	0.768	1.63(1.18-2.24)	0.003	0.066	0.137	0.636
CT/TT vs.CC	1.39(1.03-1.86)	0.029	0.046	0.099	0.547	1.16(0.91-1.47)	0.239	0.018	0.040	0.313
rs11549467										
GA vs.GG	0.76(0.54-1.06)	0.110	0.078	0.161	0.678	1.02(0.79-1.33)	0.856	0.031	0.067	0.442
AA vs.GG	1.15(0.71-1.87)	0.565	0.241	0.416	0.887	0.92(0.59-)1.42	0.697	0.182	0.334	0.846
GA/AA vs.GG	0.84(0.62-1.14)	0.264	0.056	0.119	0.597	1.00(0.78-1.28)	0.999	0.022	0.048	0.359
rs2057482										
CT vs. CC	1.32(0.97-1.81)	0.078	0.064	0.134	0.630	1.03(0.79-1.33)	0.855	0.026	0.057	0.399
TT vs. CC	2.05(1.30-3.23)	0.002	0.205	0.368	0.865	1.71(1.16-2.51)	0.007	0.128	0.248	0.784
CT/GTT vs. CC	1.46(1.09-1.94)	0.010	0.042	0.090	0.520	1.14(0.90-1.45)	0.270	0.02	0.043	0.330
rs10873142										
TC vs. TT	0.80(0.59-1.10)	0.167	0.066	0.138	0.638	0.82(0.63-1.06)	0.130	0.024	0.053	0.379
CC vs. TT	0.53(0.29-)0.96	0.037	0.061	0.128	0.617	0.68(0.44-1.04)	0.078	0.162	0.303	0.827
TC/CC vs. TT	0.74(0.55-0.99)	0.044	0.042	0.090	0.522	0.78(0.61-1.00)	0.045	0.021	0.045	0.342

*P*^a^*,* P value for multivariate Cox regression.

### Stratified analysis of HIF1A genotype effects on NSCLC prognosis

To explore the relationship between clinical characteristics, *HIF1A* polymorphisms (rs11549465 and rs2057482), and survival outcomes, we conducted stratified analyses (Tables 4, 5). Among smokers, patients with the rs11549465 TT genotype had a worse PFS than patients with the CC genotype (20.4 vs. 42.1, *P*=0.004). Multivariate Cox regression analyses confirmed the association of the TT genotype with PFS (HR=1.91, 95% CI: 1.23-2.95, *P*=0.004) and OS (HR=1.84, 95% CI: 1.25-2.70, *P*=0.002). Among non-smokers, the TT genotype was associated with PFS (39.9 vs. 74.9; HR=2.31, 95% CI: 1.08-4.97, *P*=0.032) but not OS (adjusted *P*=0.3737). The association between rs11549465 polymorphisms and PFS was not affected by the histological type (*P*<0.05). Although TT was not associated with OS (*P*=0.165) in SCC patients, it was associated with OS in patients with ADC or other lung cancer types (*P*=0.002). The TT genotype was associated with poor prognosis (PFS and OS) in patients with stage IIIA disease and those treated with chemotherapy, but not associated with OS in patients with stage IIIB disease (*P*=0.063) or those receiving chemotherapy (*P*=0.318). The radiation dose also influenced the association between rs11549465 polymorphisms and NSCLC prognosis. Among patients receiving a dose of ≥70 Gy, the TT genotype increased the risk of poor PFS (*P*<0.001) and OS (*P*=0.006). For patients receiving a dose of <70 Gy, the TT genotype was associated with PFS (HR=1.83, 95% CI: 1.09-3.04, *P*=0.021) but not OS (HR=1.47, 95% CI: 0.92-2.35, *P*=0.107).

**Table 4:** Stratified analysis of clinical parameters for rs11549465 and survival outcomes.

**Parameters**	**Subgroup**	**Genotype**	**Progression-free survival**					**Overall survival**				
			**Event/No.**	**MST**	***P***^a^	HR*(95%CI)	***P***^b^	**Event/No.**	**MST**	***P***^a^	HR*(95%CI)	***P***^b^
rs11549465												
Smoking	Yes	CC	72/165	42.1		1.00		99/165	40.7		1.00	
		CT	37/81	47.0	0.311	0.94(0.62-1.42)	0.775	48/81	46.3	0.618	0.84(0.58-1.21)	0.343
		TT	34/47	20.4	0.004	1.91(1.23-2.95)	0.004	42/47	24.5	0.068	1.84(1.25-2.70)	0.002
		CT+TT	71/128	38.6	0.046	1.24(0.88-1.75)	0.226	90/128	38.6	0.215	1.13(0.83-1.53)	0.442
	No	CC	24/133	74.9		1.00		52/133	56.3		1.00	
		CT	15/65	66.3	0.900	1.41(0.73-)2.73	0.310	28/65	56.6	0.463	1.05(0.65-1.68)	0.852
		TT	10/21	39.9	<0.001	2.31(1.08-4.97)	0.032	15/21	38.6	<0.001	1.31(0.72-2.38)	0.373
		CT+TT	25/86	60.2	0.046	1.67(0.94-2.97)	0.082	43/86	51.8	0.247	1.13(0.74-1.71)	0.582
Histology	SCC	CC	28/100	57.3		1.00		51/100	43.1		1.00	
		CT	23/61	51.7	0.206	1.47(0.83-2.61)	0.191	38/61	44.7	0.489	1.18(0.76-1.83)	0.453
		TT	22/31	24.0	<0.001	2.41(1.30-4.48)	0.006	25/31	31.1	0.030	1.43(0.86-2.37)	0.165
		CT+TT	45/92	43.4	0.005	1.79(1.09-2.94)	0.023	63/92	41.0	0.130	1.27(0.86-1.86)	0.228
	ADC and other	CC	68/198	58.1		1.00		100/198	49.1		1.00	
		CT	29/85	54.9	0.952	0.93(0.59-1.46)	0.754	38/85	53.8	0.321	0.80(0.54-1.18)	0.252
		TT	22/37	28.4	0.001	2.08(1.27-3.42)	0.004	32/37	27.6	<0.001	1.96(1.29-2.97)	0.002
		CT+TT	51/122	47.6	0.120	1.24(0.85-1.80)	0.272	70/122	45.2	0.357	1.10(0.80-1.51)	0.222
Stage	IIIA	CC	28/143	71.7		1.00		52/143	57.2		1.00	
		CT	21/65	51.7	0.066	1.79(1.00-3.20)	0.051	27/65	60.2	0.851	1.09(0.67-1.75)	0.734
		TT	9/19	37.7	0.002	3.76(1.65-8.57)	0.002	14/19	31.4	0.001	2.95(1.53-5.70)	0.001
		CT+TT	30/84	51.7	0.009	2.08(1.21-3.55)	0.008	41/84	54.3	0.171	1.35(0.88-2.07)	0.172
	IIIB	CC	68/155	44.6		1.00		99/155	38.8		1.00	
		CT	31/81	55.8	0.586	0.82(0.53-1.27)	0.368	49/81	43.9	0.583	0.89(0.62-1.27)	0.507
		TT	35/49	22.6	0.001	1.73(1.14-2.65)	0.011	43/49	29.0	0.023	1.42(0.98-2.05)	0.063
		CT+TT	66/130	43.6	0.182	1.14(0.80-1.63)	0.455	92/130	38.2	0.447	1.08(0.81-1.46)	0.593
Chemotherapy	No	CC	54/187	63.4		1.00		90/187	50.5		1.00	
		CT	29/79	56.9	0.137	1.55(0.98-2.46)	0.063	38/79	52.4	0.994	1.06(0.72-)1.57	0.767
		TT	25/39	23.7	<0.001	2.94(1.78-4.87)	<0.001	33/39	27.9	<0.001	2.07(1.35-3.17)	0.001
		CT+TT	54/118	47.7	0.001	1.97(1.34-2.91)	0.001	71/118	43.5	0.047	1.37(0.99-1.88)	0.057
	Yes	CC	42/111	48.1		1.00		61/111	39.8		1.00	
		CT	23/67	51.9	0.479	0.72(0.42-1.22)	0.223	38/67	48.1	0.530	0.79(0.51-1.20)	0.264
		TT	19/29	30.0	0.038	1.22(0.69-2.17)	0.492	24/29	30.8	0.132	1.29(0.79-2.11)	0. 318
		CT+TT	42/96	44.9	0.670	0.88(0.56-1.39)	0.591	62/96	43.7	0.858	0.93(0.64-1.34)	0.684
Dose	≥70Gy	CC	34/150	69.7		1.00		65/150	53.0		1.00	
		CT	26/85	63.0	0.136	1.36(0.80-2.32)	0.252	39/85	53.1	0.954	0.94(0.62-1.42)	0.938
		TT	20/34	24.8	<0.001	3.01(1.67-5.41)	<0.001	30/34	28.8	<0.001	1.91(1.20-3.01)	0.006
		CT+TT	46/119	53.7	0.002	1.77(1.11-2.83)	0.016	69/119	44.8	0.088	1.19(0.84-1.71)	0.331
	<70Gy	CC	62/148	42.5		1.00		86/148	39.2		1.00	
		CT	26/61	44.5	0.995	0.98(0.61-1.58)	0.983	37/61	46.8	0.906	0.99(0.66-1.47)	0.944
		TT	24/34	26.9	0.003	1.83(1.09-3.04)	0.021	27/34	29.6	0.030	1.47(0.92-2.35)	0.107
		CT+TT	50/95	39.0	0.141	1.25(0.85-1.84)	0.265	64/95	42.3	0.323	1.14(0.81-1.60)	0.461

*P^a^, Log-rank P; *P*^b^, cox regression; SCC, squamous cell carcinoma; ADC, adenocarcinoma; MST, mean survival time.

**Table 5:** Stratified analysis of clinical parameters for rs2057482 and survival outcomes.

**Parameters**	**Subgroup**	**Genotype**	**Progression-free survival**					**Overall survival**				
			**Event/No.**	**MST**	***P***^a^	HR*(95%CI)	***P***^b^	**Event/No.**	**MST**	***P***^a^	HR*(95%CI)	***P***^b^
rs2057482												
Smoking	Yes	CC	75/178	48.9		1.00		107/178	43.4		1.00	
		CT	53/93	36.6	0.062	1.31(0.91-1.87)	0.147	61/93	39.2	0.916	1.00(0.72-1.38)	0.998
		TT	15/22	20.1	0.023	1.48(0.84-2.62)	0.173	21/22	26.6	0.027	1.55(0.96-2.52)	0.075
		CT+TT	68/115	35.0	0.018	1.34(0.96-1.88)	0.087	82/118	36.6	0.390	1.10(0.82-1.48)	0.541
	No	CC	25/136	75.0		1.00		54/136	43.4		1.00	
		CT	15/68	65.2	0.710	1.31(0.67-2.54)	0.434	29/68	39.2	0.900	1.08(0.67-1.73)	0.763
		TT	9/15	19.0	<0.001	4.65(1.99-10.89)	<0.001	12/15	26.6	0.009	2.40(1.22-4.73)	0.012
		CT+TT	24/83	57.9	0.131	1.77(0.98-3.18)	0.058	41/83	52.9	0.455	1.29(0.84-1.97)	0.247
Histology	SCC	CC	29/111	68.2		1.00		59/111	46.7		1.00	
		CT	32/64	36.3	0.002	2.43(1.43-4.13)	0.001	39/64	43.5	0.390	1.31(0.86-1.99)	0.206
		TT	12/17	14.9	<0.001	4.09(1.99-8.43)	<0.001	16/17	23.1	<0.001	2.71(1.49-4.93)	0.001
		CT+TT	44/81	33.1	<0.001	2.71(1.65-4.46)	<0.001	55/81	38.4	0.059	1.53(1.04-2.24)	0.032
	ADC and other	CC	71/203	56.3		1.00		102/203	48.9		1.00	
		CT	36/97	54.5	0.875	0.90(0.60-1.36)	0.624	51/97	19.2	0.534	0.89(0.63-1.25)	0.505
		TT	12/20	22.7	0.045	1.47(0.78-2.76)	0.237	17/20	33.4	0.100	1.29(0.76-2.19)	0.354
		CT+TT	48/117	50.4	0.633	1.00(0.69-1.45)	0.992	68/117	45.6	0.990	0.97(0.71-1.32)	0.822
Stage	IIIA	CC	28/132	68.1		1.00		47/132	58.6		1.00	
		CT	21/79	54.2	0.492	1.19(0.67-2.11)	0.561	32/79	59.4	0.974	0.93(0.59-1.47)	0.757
		TT	9/16	21.6	<0.001	4.45(1.93-10.22)	<0.001	14/16	27.0	<0.001	2.56(1.34-4.88)	0.004
		CT+TT	30/95	50.1	0.094	1.52(0.90-2.58)	0.118	46/95	53.2	0.251	1.15(0.76-1.75)	0.512
	IIIB	CC	72/182	55.6		1.00		114/182	42.2		1.00	
		CT	47/82	37.9	0.033	1.34(0.92-1.96)	0.131	58/82	38.9	0.559	1.07(0.77-1.48)	0.688
		TT	15/21	18.6	0.028	1.56(0.88-2.75)	0.127	19/21	29.7	0.170	1.35(0.82-2.23)	0.239
		CT+TT	62/103	35.2	0.009	1.39(0.98-1.97)	0.065	77/103	36.5	0.317	1.13(0.84-1.52)	0.430
Chemotherapy	No	CC	62/194	60.6		1.00		97/194	49.1		1.00	
		CT	38/95	51.4	0.363	1.22(0.81-1.84)	0.350	49/95	49.9	0.593	1.00(0.70-1.42)	0.997
		TT	8/16	18.9	0.032	1.70(0.80-3.62)	0.170	15/16	25.8	0.001	2.28(1.31-3.98)	0.004
		CT+TT	46/111	49.6	0.165	1.28(0.87-1.89)	0.214	64/111	45.6	0.684	1.16(0.84-1.60)	0.380
	Yes	CC	38/120	57.0		1.00		64/120	46.7		1.00	
		CT	30/66	43.6	0.105	1.37(0.83-2.25)	0.217	41/66	42.2	0.580	1.05(0.70-1.58)	0.804
		TT	16/21	19.4	0.001	2.41(1.31-4.42)	0.005	18/21	30.7	0.126	1.43(0.83-2.48)	0.197
		CT+TT	46/87	37.2	0.011	1.62(1.04-2.53)	0.032	59/87	38.6	0.269	1.14(0.79-1.65)	0.480
Dose	≥70Gy	CC	42/169	67.3		1.00		72/169	54.6		1.00	
		CT	30/86	56.5	0.106	1.53(0.95-2.48)	0.080	49/86	45.2	0.142	1.51(1.04-)2.19	0.029
		TT	8/14	21.4	0.003	2.70(1.22-5.98)	0.014	13/14	29.1	0.001	2.61(1.42-4.93)	0.002
		CT+TT	38/100	52.6	0.026	1.68(1.07-2.63)	0.025	62/100	41.4	0.025	1.65(1.17-)2.34	0.004
	<70Gy	CC	58/145	50.1		1.00		89/145	39.6		1.00	
		CT	38/75	41.1	0.448	1.17(0.77-1.78)	0.467	41/75	50.9	0.119	0.69(0.47-1.01)	0.059
		TT	16/23	18.4	0.016	1.75(0.99-3.10)	0.054	20/23	27.5	0.153	1.38(0.83-2.30)	0.215
		CT+TT	54/98	37.2	0.131	1.30(0.89-1.90)	0.169	61/98	45.4	0.443	0.83(0.59-1.16)	0.273

*P^a^, Log-rank P; *P*^b^, multivariate Cox regression; SCC, squamous cell carcinoma; ADC, adenocarcinoma; MST, mean survival time; MST, mean survival time.

Smoking did not influence the association between rs2057482 polymorphisms and survival outcomes (PFS and OS). The TT genotype was associated with PFS and OS in both smokers and non-smokers. In SCC patients, the TT genotype was associated with a poor PFS (HR=1.09, 95% CI:1.99-8.43, *P*<0.001) and OS (HR=2.71, 95% CI:1.49-4.93, *P*=0.001). These associations were not significant in patients with other histological types. The disease stage also affected the association between rs2057482 polymorphisms and prognosis. For instance, patients with stage IIIA disease had the TT genotype associated with PFS (*P*<0.001) and OS (*P*=0.004); These associations were not significant in patients with stage IIIB disease. Patients that did not receive chemotherapy had a poorer OS (HR=2.28, 95% CI: 1.31-3.98, *P*=0.004) compared to those receiving chemotherapy. In contrast, patients receiving chemotherapy had a worse PFS (HR=2.41, 95% CI: 1.31-4.42, *P*=0.005). For patients receiving a dose of ≥70 Gy, the TT genotype was associated with a poor PFS (HR=2.70, 95% CI: 1.22-5.98, *P*=0.014) and OS (HR=2.61, 95% CI: 1.42-4.93, *P*=0.002). For patients receiving a dose of<70Gy, t However, none of genotypes were associated with survival.

### The association between the SNPs and HIF1A mRNA levels

**Figure 5:**
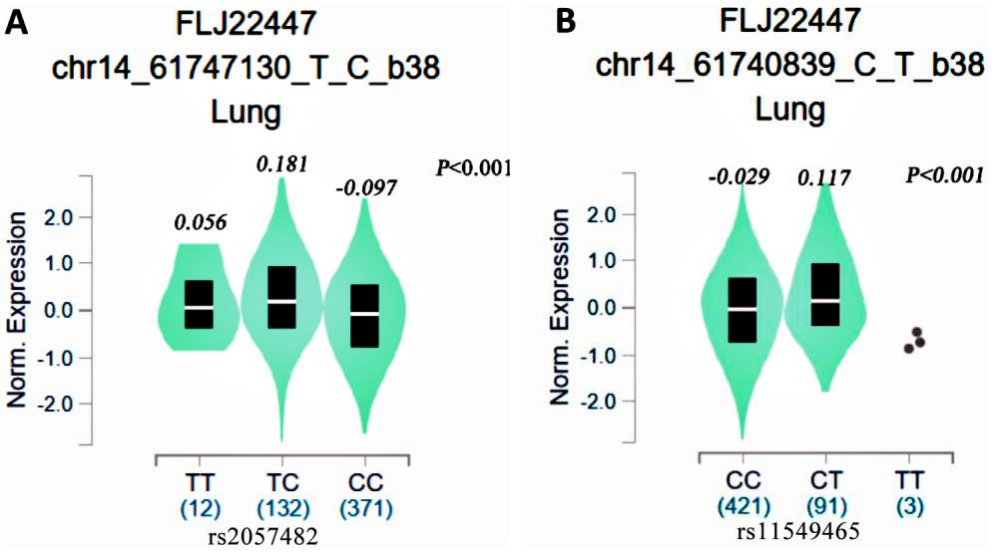
eQTL analysis of HIF1-alpha SNPs from the GTEx database (**A**: rs2057482, **B**: 11549465).

We used the GTEx database to assess the relationships between the rs2057482 and rs11549465 SNPs and *HIF1A* mRNA levels. There were significant differences in mRNA expression among the three genotypes. *HIF1A* mRNA levels were elevated in lung tissues harboring the rs2057482 (Figure 5A) or rs11549465 T allele (Figure 5B). By contrast, the C allele was associated with *HIF1A* downregulation.

## DISCUSSION

We identified two *HIF1A* SNPs (rs11549465 and rs2057482) that were associated with the survival of NSCLC patients undergoing radiotherapy. Individuals with the rs11549465 and rs2057482 T alleles had a worse PFS and OS than patients with the corresponding C allele. Stratified analyses indicated that smoking, histological type, TNM stage, chemotherapy, and radiation dose were factors affecting the association between *HIF1A* SNPs and OS in NSCLC patients. However, these factors had no effect on the relationship between *HIF1A* SNPs and PFS. We also found that the T alleles of rs11549465 and rs2057482 were associated with elevated *HIF1A* mRNA levels. To the best of our knowledge, this is the first study to show an association between *HIF1A* polymorphisms and the prognosis of locally advanced NSCLC in patients undergoing radiation therapy.

The human *HIF1* gene is located on chromosome 14 (14q21-24). The 3,720-bp sequence of *HIF1A* encodes 826 amino acids. Under hypoxic conditions, HIF-1 accumulates in the cells upon prolyl-hydroxylation failure at positions 402 and 564. Subsequently, the transcriptionally active HIF-1 complex binds to DNA sequences of downstream target genes, including vascular endothelial growth factor (VEGF), erythropoietin, inducible nitric oxide synthase, heme oxygenase 1, and glycosylase [17]. Expression of these genes has been linked to lung cancer development, progression, and metastasis. Elevated HIF-1α levels have also been associated with lung cancer development and progression [18]. Previous studies have shown that the rs2057482 T allele plays a protective role in cervical cancer. Notably, individuals with the CC genotype were shown to have a 1.44-fold higher risk of lung cancer than those with CT/TT genotypes [19]. The rs2057482 T allele has also increases the risk of prostate cancer in the Chinese Han population [20]. He et al. found that the TT genotype of *HIF1A* was common in small cell carcinoma (OR=1.73, 95% CI: 1.05-2.84) and that among passive smokers, rs2057482 TT carriers were 2.195 times more likely to develop lung cancer compared to CC carriers (95% CI: 1.038-4.463). Guo et al. found that hepatocellular carcinoma patients with the *HIF1A* rs2057482 TT genotype had a worse prognosis compared to those with the CC genotype [21], which contradicts our findings. These discrepancies suggest that the polymorphisms effects on patient survival may be tumor type-specific. Additionally, each *HIF1A* SNP may have a different effect on prognosis. For instance, the rs2057482 TT genotype could play a protective role in hepatocellular carcinoma but lead to a poor prognosis in lung cancer. Wu et al. investigated the effects of *HIF1A* genetic variants and *A* mRNA levels on chemotherapy response and patient survival. They found that the *C1772T (P582S) CC* genotype was associated with a chemotherapy response and that patients with the TT genotype had a poor OS and PFS [14], consistent with our findings. Our results also suggest that the T alleles of rs2057482 and rs11549465 are risk factors associated with poor NSCLC prognosis.

Radiation therapy is one of the most common treatments for locally advanced tumors. Radiation can induce HIF-1 activity in tumor cells, which causes an upregulation of VEGF and b fibroblast growth factor. These cytokines reduce the endothelial cell sensitivity to radiation, thereby promoting radiotherapy resistance in tumors. Thus, elevated HIF-1 levels may enhance radiation resistance in tumors [22]. Sensitivity to radiotherapy may be restored by inhibiting HIF-1 expression or activity. For instance, Kessler et al. used short-interfering RNAs (siRNAs) to suppress HIF-1 expression in human glioma cells (U251 and U343) and found that under hypoxic conditions the radiation resistance of glioma cells was alleviated [23]. In refractory ovarian cancer, siRNAs were used to downregulate HIF-1 and its downstream gene *VEGF*. Inhibition of HIF-1 and VEGF expression reduced tumor cell proliferation rate [24]. HIF-1 also regulates microRNAs expressions. For example, under hypoxic conditions, HIF-1 induces the expression of mir-210, mir-155, mir-372/373, and mir-10b, as well as downregulates the expression of mir-20b and mir-200b. In hypoxic human liver cancer cells (SMMC-7721, HepG2, and HuH7), downregulation of mir-210 inhibited cancer cell proliferation, induced cell apoptosis, and enhanced radiation sensitivity [25].

HIF-1α inhibits cancer cell apoptosis by promoting the expression of VEGF, glucose transporter 1, and other anti-apoptotic agents [26]. Additionally, HIF-1 enhances the expression of VEGFR2, TGFβ, and endothelin-1, all of which induce neovascularization and increase vascular permeability [27]. Under hypoxic conditions, lung cancer cells produce most of their ATP through glycolysis [28]. Here, we showed significant differences in *HIF1A* mRNA levels among patients with different genotypes. Polymorphisms may affect the transcription of *HIF1A* and thereby regulate HIF-1 protein levels and activity. Future studies are needed to confirm the functional roles of *HIF1A* polymorphisms. Given the crucial role of HIF-1 in angiogenesis, metabolism, and DNA repair, polymorphisms affecting *HIF1A* may provide an advantage to tumor cells by resisting the cytotoxic effects of radiation.

In the stratified analysis, we noted that the gene polymorphisms affected the prognosis of patients with smoking status, histology, stage, chemotherapy, and radiation dose. Broadly speaking, smoking, histology, stage, and dose did not change the effect of rs11549465 polymorphism on lung cancer prognosis. However, patients without chemotherapy and rs11549465 TT had a worse prognosis. This suggests that interactions exist between the genes and chemotherapy. Similar results were not found for rs2057482. Taken together, these findings indicate that the prognosis was affected by gene factors and the environment.

This study has several limitations. First, it was a single-center study, and selection bias could have affected our findings. Future multi-center studies are required to confirm the findings presented here. Second, we found that the rs11873142 C allele was weakly associated with poor OS. The FPRP analysis produced a *P*-value of <0.2, possibly reflecting the low statistical power of this analysis. The associations also need to be verified in a s larger cohort size. Third, we selected SNPs that are common among the Asian population. Thus, the functional roles of other SNPs should be investigated. Additionally, although the results showed an expression trend that was caused by different genotypes, the data from eQTL needs to be verified using a larger sample size. Finally, our analyses were limited to a single gene, and future studies should determine the association of multiple genes with survival outcomes in NSCLC patients.

In conclusion, we identified *HIF1A* SNPs rs11549465 and rs2057482 as associated with survival in NSCLC patients undergoing radiation therapy. These associations were affected by different clinical characteristics. Hence, these two SNPs can be independent prognostic biomarkers in NSCLC patients undergoing radiation therapy. However, the prognostic value of these SNPs should be validated in large-cohort, prospective studies.

## MATERIALS AND METHODS

### Study population

This was a single-center follow-up study. All patients were newly diagnosed with NSCLC as confirmed by pathological examination and treated with radiotherapy or chemoradiotherapy. The tumor stage was determined from the American Joint Committee on Cancer/Union for International Cancer Control (AJCC/UICC 7^th^ edition) guidelines [29]. The histological grade was determined by the World Health Organization classification system for NSCLC. Patients that had other tumors, a cancer history of, underwent surgery or stereotactic ablative radiotherapy, were excluded from the study. Patients with severe immune diseases, inflammatory diseases, severe organ failure, cardiovascular diseases, or a life expectancy of less than one month were also excluded. The study was approved by the Ethics Committee of the Second Affiliated Hospital of Zhengzhou University.

### SNP identification and genotyping

To identify *HIF1A* SNPs, we searched the dbSNP database (https://www.ncbi.nlm.nih.gov/snp/) for the *HIF1A* gene with the minor allele frequency set to >3%. Subsequently, we analyzed the linkage disequilibrium of the identified SNPs using the 1000 Genomes Project database. We searched PubMed and the Chinese China National Knowledge Infrastructure to identify studies of the association between HIF1 gene polymorphisms and lung cancer risk among Chinese populations. We analyzed the search results for overlaps inrs2057482, rs11549465, rs10873142, rs11549467, rs2301113, rs41508050, rs10645014, rs41492849, rs34005929; and identified the following four SNPs: rs11549465, rs11549467, rs2057482, rs10873142.

To genotype the SNPs, we extracted the genomic DNA from patients using 5 ml of venous blood. SNPs were genotyped using the polymerase chain reaction (PCR)-based restriction fragment length polymorphism method and the TaqMan SNP Genotyping Assays Kit (Thermo Fisher Scientific, USA). DNA samples were stored at −20° C, and DNA amplification was performed separately for the four variants using predesigned reverse and forward primers (Supplementary Table 1). Amplification of the exon 12 region (rs11549465, rs11549467) of *HIF1A* was performed in a final volume of 50 μl containing 50 ng of genomic DNA, 2.5 mM MgCl_2_, 100 mM dNTP, 50 pmol/μl of each primer, and 1.0 U/ml of Taq DNA polymerase. For rs2057482 and rs10873142, the 50-μl reaction mixture contained 50 ng of template DNA, 2.5 mM MgCl_2_, 100 pmol/μl of each primer, 100 mM dNTP, and 1 U/μl Taq DNA polymerase. Amplification was performed at 95° C for 10 min, followed by 40 cycles of 95° C for 15 sec and 60° C for 1 min. The final extension was performed at 72° C for 10 min. PCR products were resolved by 2% agarose gel electrophoresis, and the results were analyzed using TYPER 4.0. For the rs11549465 polymorphism, the C allele yielded 128-bp and 19-bp products, and the T allele yielded a 147-bp product. For the rs11549467 polymorphism, the G allele yielded 143-bp and 114-bp products, and the A allele yielded a 255-bp product. For the rs2057482 polymorphism, the restriction fragments were 191 bp and 145 bp. For the rs10873142 polymorphism, the restriction fragments were 864 bp for the T allele and 707 bp and 157 bp for the C allele.

### Data collection

The clinical characteristics of patients were obtained from medical records. Data was collected for gender, age, body mass index (BMI), smoking status, drinking status, KPS), histological type, TNM stage, chemotherapy, radiation technology, and radiation dose. Current smokers or individuals with a history of daily smoking were regarded as smokers. Drinkers were those who consumed alcohol more than twice a month [30]. Individuals with a BMI >24 were considered as overweight. Patients older than >60 years were considered elderly [31, 32].

Patients were treated with routine 3-dimensional conformal radiotherapy (3D-CRT) or intensity-modulated radiotherapy (IMRT). Conventional radiotherapy targeted the primary site, ipsilateral hilum, and mediastinal drainage area. Computed tomography (CT) simulation-based positioning was performed for 3D-CRT or IMRT patients. CT and positron emission tomography (PET)/CT examination were used to assess the presence of mediastinal lymph node metastasis, and lung lesions in the lung tissue window were recorded as the gross target volume. The clinical target volume (CTV) included the ipsilateral hilum and high-risk lymph drainage area. To account for positioning errors, organ movements, and other errors, we defined the planning target volume as 5RAM around the CTV. Radiation therapy was adjusted according to the diagnosis, treatment capabilities of the radiotherapy department, and the patient’s physical strength, age, lung function, pulmonary complications, and tolerance. The treatment plan was reviewed and approved by a physician; A Varian linear accelerator was used for all treatments.

The primary outcomes were progression-free survival (PFS) and OS. PFS was defined as the period from treatment completion to death or disease progression; OS was defined as the period from treatment completion to death regardless of the cause [33]. Patient follow-up was performed through phone calls and outpatient records every three months for the first two years and every six months thereafter. CT, PET/CT, and other examinations were performed when necessary during the follow-up period.

### Expression quantitative trait loci analysis

We assessed the correlation between *HIF1A* SNPs and mRNA levels from the GTEx portal database (https://www.gtexportal.org/home/), and the results are presented as violin plots.

### Statistical analysis

Patient age and radiation doses were transformed into categorical variables. The Kaplan-Meier method and log-rank test were used to compare the PFS and OS of patients with different genotypes. Univariate and multivariate Cox regression analyses were conducted to calculate hazard risks (HRs) and 95% confidence intervals (CIs) to assess the association between clinical parameters, genotypes, and survival outcomes. The multivariate Cox regression model was adjusted for the following variables: sex (male vs. female), age (≥60 vs. <60), BMI (overweight vs. normal), smoking (yes vs. no), drinking (yes vs. no), KPS (≥80 vs. <80), histological type (squamous cell carcinoma [SSC], adenocarcinoma [ADC], or other), radiation technology (IMRT vs. CRT or other), and radiation dose (≥70 vs. <70). Multiple comparisons were corrected using false-positive report probability (FPRP) analysis with a prior probability of 0.01 to detect an HR of 1.2. Stratified analyses were performed for histological type, radiation technology, smoking, radiation dose, TNM stage, and chemotherapy. All analyses were conducted using SPSS 23.0 and GraphPad Prism 8.0 software. A value of *P*<0.05 was considered significant unless specified otherwise.

### Data accessibility

The original data is available from the corresponding authors upon request.

## Supplementary Material

Supplementary Table 1
