# Supplementary material for: Association of HIF1-α gene polymorphisms with advanced non-small cell lung cancer prognosis in patients receiving radiation therapy
Source: Aging (Albany NY). 2021 Feb 17;13(5):6849–65. doi: 10.18632/aging.202542 (PMC7993740; doi:10.18632/aging.202542)
Supplement: Supplementary Table 1 [file aging-13-202542-s001.pdf]

SUPPLEMENTARY TABLE

Supplementary Table 1. Primers and digestion condition for PCR-RFLP.

SNPs	Primers (5'-3')
rs11549165	FP: AAGGTGTGGCCATTGTAA AAACTC
	RP: GCACTAGTAGTTTCTTTATGTATG
rs11549467	FP: AAGGTGTGGCCATTGTAA AAACTC
	RP: GCACTAGTAGTTTCTTTATGTATG
rs2057482	FP: CGCAAGTCCTCAAAGCACA
	RP: TCAGTGGTGGCAGTGG TAGT
rs10873142	FP: TTCTGTTCCTGGGTTATCTCA
	RP: CCTTTAATGCAACAATGCCTAC
